# Reversal of phenotypic resistance in multi-drug resistant carbapenemase-producing *K. pneumoniae* clinical isolates due to *in vitro* synergistic interactions between bacteriophages and antibiotics at clinically achievable concentrations

**DOI:** 10.1093/jac/dkaf163

**Published:** 2025-05-31

**Authors:** Paschalis Paranos, Maria Siopi, Eleni Papanikolaou, Sophia Vourli, Panagoula Kolia, Spyros Pournaras, Joseph Meletiadis

**Affiliations:** Clinical Microbiology Laboratory, Attikon University Hospital, Medical School, National and Kapodistrian University of Athens, Athens, Greece; Clinical Microbiology Laboratory, Attikon University Hospital, Medical School, National and Kapodistrian University of Athens, Athens, Greece; Department of Human Genetics, Faculty of Biology, National and Kapodistrian University of Athens, Athens, Greece; Clinical Microbiology Laboratory, Attikon University Hospital, Medical School, National and Kapodistrian University of Athens, Athens, Greece; Department of Human Genetics, Faculty of Biology, National and Kapodistrian University of Athens, Athens, Greece; Clinical Microbiology Laboratory, Attikon University Hospital, Medical School, National and Kapodistrian University of Athens, Athens, Greece; Clinical Microbiology Laboratory, Attikon University Hospital, Medical School, National and Kapodistrian University of Athens, Athens, Greece

## Abstract

**Background:**

Therapeutic options for MDR carbapenemase-producing *Klebsiella pneumoniae* (CRKP) are limited. We therefore assessed the *in vitro* activity of five antibiotics from different classes in combination with lytic bacteriophages (phages) against MDR CRKP isolates.

**Material and methods:**

A total of 15 non-repetitive, well-characterized MDR CRKP isolates and four phages belonging to the *Podoviridae* family were used in chequerboard assays with amikacin, meropenem, ciprofloxacin, colistin and ceftazidime/avibactam. The spectrophotometrically determined MIC of drugs and phages alone and in combination were used to calculate the fractional inhibitory concentration index (FICi). The clinical relevance was assessed based on the MIC reductions at clinically achievable concentrations and below the corresponding susceptibility breakpoints. Emergence of resistance was studied in growth curves and time–kill experiments.

**Results:**

Synergy was found for ciprofloxacin in 6/15 (40%) isolates, meropenem in 10/15 (67%), ceftazidime/avibactam in 11/15 (73%), colistin in 8/15 (53%) and amikacin in 9/15 (60%) with all four phages against host bacteria. The synergistic interactions were strong as the FICi were 0.01–0.35 reducing the MICs (>90% growth inhibition) to clinically achievable concentrations for 87%–100% of strains, except ciprofloxacin. Reversal of phenotypic resistance was observed for amikacin, meropenem, colistin and ceftazidime/avibactam in 100%, 53%, 89% and 80% of isolates, respectively. No emergence of resistance was found for isolates with low level resistance to amikacin (MΙC 64 mg/L).

**Conclusions:**

The phage–antibiotic combinations were synergistic against more than half of the isolates for all antibiotics except ciprofloxacin reversing resistance in most strains particularly with amikacin.

## Introduction

*Klebsiella pneumoniae* is an encapsulated Gram-negative, opportunistic bacterium that can cause wide range of complex recurrent infections, particularly in immunocompromised individuals with high rates of morbidity and mortality.^1^ Carbapenem-resistant *K. pneumoniae* (CRKP) is a looming threat that is increasingly reported worldwide. According to ECDC, in Greece 72% of the isolates were CRKP, making it having the highest percentage rate among the other European countries (https://atlas.ecdc.europa.eu/public/index.aspx). According to a recent study, a considerable proportion (74.3%) of CRKP isolates had MDR phenotype whereas a 25.7% were either extensively or pan-drug resistant.^2^ For that reason, taking decisive actions as well as introducing new therapies in our armamentarium is of primary importance for the fight against CRKP isolates.

A potential alternative therapeutic approach for treating infections caused by CRKP isolates is phage therapy.^3^ Despite the fact that phages have been used as an anti-infective agent for more than a century, the rising rates of antimicrobial resistance renewed interest in combatting antibiotic-resistant bacteria in recent years.^4^ However, bacteria develop rapidly resistance to phages and this is the major limitation of phage therapy and particularly of monophage therapy.^5^ As resistance mechanisms to phages may differ from those against antibiotics, one approach to overcome resistance to both agents is combination therapy with phages and antibiotics.^4^ Antibiotics are considered the current standard of care and using phages as an adjuvant therapy is a valid way to overcome bacterial resistance, enhance efficacy, broaden the spectrum of therapy, clear the infection faster and reach difficult to treat sites of infection.^6,7^ Despite, the extensive research that has been performed over recent years around the synergistic interaction of phages in combination with antibiotics, these studies focus on biofilm and species other than *K. pneumoniae* and particularly CPKP.^6^

We therefore investigated the interactions between five different classes of antibacterial agents (colistin, meropenem, ciprofloxacin, amikacin, ceftazidime/avibactam) and different phages against MDR CRKP isolates.

## Material and methods

### Bacterial strains

A total of 15 non-repetitive, well-characterized metallo-β-lactamase (MBL)-producing *K. pneumoniae* isolates (five *bla*_NDM,_ five *bla*_VIM_ and five *bla*_VIM_ and *bla*_KPC_) with resistance profiles to carbapenems, cephalosporins and fluoroquinolones (100%) and aminoglycosides (53%), were used.^8^ MLST analysis as well as identification of *K* loci was performed on all 15 isolates by web-based methods based on whole-genome sequencing data.^9,10^ The strains were stored at −80°C in trypticase soy broth supplemented with 20% glycerol until further use. Before testing, the isolates were revived by subculturing them onto MacConkey agar No. 3 (MAC) (Oxoid, Athens, Greece) plates at 37°C for 24 h.

### Bacteriophages

Four phages (vB_KpP_AttikonH1, vB_KpP_AttikonH2, vB_KpP_AttikonH4, vB_KpP_AttikonH5) specific against MDR *K. pneumoniae* isolates, which belong to the *Podoviridae* family, were previously isolated from influent wastewater samples collected from Athens’ central wastewater plant in Psyttaleia.^11^ The isolated phages were purified by triplicate single plaque transfer and propagated until homologous plaques were obtained. Their titres were determined by single-layer agar (SLA) plaque assay^12^ and their stocks were kept in Luria-Bertani medium (PanReac Applichem, Athens, Greece) at 4°C until further use. The phages presented in this study are available in the GenBank nucleotide sequence database under accession numbers PP974225, PP978610, PP978611 and PP978613.

### Drugs and medium

Laboratory-grade standard powders of amikacin, meropenem, ciprofloxacin, colistin, ceftazidime/avibactam (Sigma-Aldrich, Athens, Greece) and colistin (Pfizer Inc., PA, USA) were dissolved according to the manufacturer’s instructions, and stock solutions of 40× for water soluble and 400× for water insoluble drugs were stored in aliquots at −70°C. The medium used throughout was cation-adjusted Mueller–Hinton broth II (caMHB) supplemented with 25 mg/L calcium and 12.5 mg/L magnesium (Difco, Athens, Greece).

### In vitro *susceptibility testing*

The minimal inhibitory concentrations of antibiotics (MIC_A_) were determined according to the ISO-standard broth microdilution method.^13^ Briefly, 2-fold serial drug concentrations were prepared in caMHB in order to yield 2× the final concentrations, which ranged from 256 to 4 mg/L for amikacin, meropenem and ciprofloxacin, 8 to 0.125 mg/L for colistin, and 64/4 to 1/4 mg/L for ceftazidime/avibactam. Then 50 μL of each drug concentration and a drug-free growth control (50 μL of assay medium) were dispensed into sterile, flat-bottom, tissue-treated 96-well plates (Nunc^™^ MicroWell^™^ 96-Well, ThermoFisher). Once the microdilution trays were prepared, they were stored at −80°C until use for up to 1 month. On the day of the experiment, they were thawed, inoculated with 50 μL of a 5 × 10^5^ cfu/mL bacterial suspension prepared from a 24 h culture grown on MAC and incubated at 35 ± 2°C in ambient air for 18–24 h. Bacterial CFU count was confirmed via quantitative cultures of serial 10-fold dilution. The MICs were assessed spectrophotometrically at OD_550_ after agitation (220 rpm, 1 min) as the lowest drug concentration resulting in >90% inhibition of bacterial growth. Three independent replicates were assessed between different days and the median (range) MIC was calculated. The recommended *Escherichia coli* ATCC 25922 and *Pseudomonas aeruginosa* ATCC 27853 were used as quality control strains.

The minimum inhibitory concentrations of phages (MIC_P_) were determined by a broth microdilution method. In short, phages were serially diluted 1:10 in caMHB and 50 μL were placed in each well of 96-well plates in order to reach final phage concentrations 5 × 10^9^–5 × 10^0 ^PFU/mL (MOI 10^4^–10^−5^). Phage concentrations were quantitated using SLA spot of serial 10-fold dilution. Moreover, a negative and a growth control were used for each isolate and phage combination. Bacterial inoculum was prepared from a 24 h culture grown on a MAC using 0.5 McFarland standard and 50 μL of 1:100 dilution in caMHB were placed in the plate, with already inoculated phages, resulting in a concentration of 5 × 10^5^ cfu/mL. Three independent replicates were performed on different days and the median (range) MIC_P_ was calculated. Plates were incubated at 35 ± 2°C for 24 h and MIC_P_s were assessed spectrophotometrically at OD_550_ after agitation (220 rpm, 1 min) as the lowest phage concentration resulting in >90% inhibition of bacterial growth.

### In vitro *combination testing*

**Figure 1.**
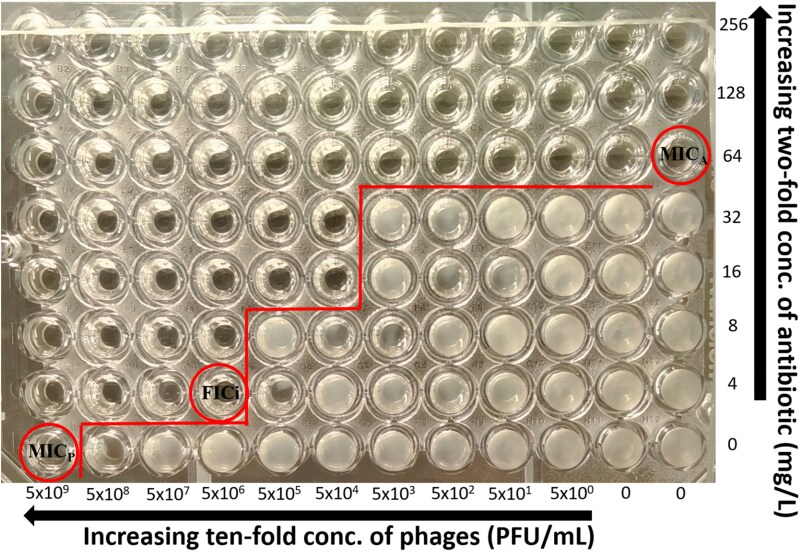
Meropenem + phage combination against MDR carbapenemase-producing *K. pneumoniae* in the broth microdilution chequerboard method. Meropenem MIC (MIC_A_) and phage MIC alone (MIC_P_) were 64 mg/L and 5 × 10^9^ PFU/mL, respectively. When they were combined, a synergistic interaction was observed in conjunction with reversal of phenotypic resistance for meropenem as the MIC of meropenem was reduced from 64 mg/L to 4 mg/L, below the resistance breakpoint (8 mg/L), and the MIC of phage was reduced from 5 × 10^9^ PFU/mL to 5 × 10^6^ PFU/mL. The fractional inhibitory concentration index (FICi) was 0.06 (red circle).

The antibiotic-phage interactions were determined using a two-dimensional chequerboard assay.^14^ Briefly, antibacterial drugs were 2-fold serially diluted in caMHB to yield 4× the aforementioned final concentrations and 25 μL of each drug concentration were dispensed to consecutive wells to each row (A-G) in a 96-well plate (ThermoFisher, cat. no. 167008) at concentrations 4× higher than those finally tested after addition of 25 μL of phages at 4× the final concentration and 50 μL of bacterial suspension of 2× the final inoculum as described below and placed at −70°C. Phages were serially diluted 1:10 in 96 deep-well plates in caMHB to have final concentrations in the plate of 5 × 10^9^–5 × 10^0 ^PFU/mL. On the day of the experiment, plates were thawed properly at room temperature and 25 μL of phage suspension were added in each well of the 96-well microplate. Each plate consisted of 2-fold decreasing concentrations of the drug in rows A to G with row H containing no drug and 10-fold decreasing concentrations of phages in columns 1 to 11 with column 12 containing no phage (Figure 1). Well H12 was used as a growth control, while a sterility control was placed in blood agar in every experiment. A bacterial suspension of 0.5 McFarland concentration was prepared from a 24 h culture and 50 µL of a 1:100 dilution in caMHB was added to the wells of the plate, which were already inoculated with phage, resulting in a concentration of 5 × 10^5^ cfu/mL. Three independent replicates were assessed between different days and the median (range) MIC_A_ and MIC_P_ was calculated. The plates were incubated at 35 ± 2°C for 24 h and the MIC_A_s and MIC_P_s alone and in combination were determined spectrophotometrically at OD_550_ after agitation (220 rpm) as the lowest concentration with >90% inhibition of bacterial growth. Growth found in single wells were excluded from the analysis based on the assumption that any interaction pattern should cover multiple wells.

### Antibiotic–phage interaction analysis

The FICi was calculated using the following equation for each antibiotic (A)–phage (P) combination:


$$FICi=FIC_{A}+FIC_{P}=\frac{MIC_{A+P}}{MIC_{A}\;alone}+\frac{MIC_{P+A}}{MIC_{P}\;alone}$$

where $\mathrm{MI}C_{A}\;\mathrm{alone}$ and $\mathrm{MI}C_{P}\;\mathrm{alone}$ is the MIC of drug alone and the MIC of phage alone, respectively, and $\mathrm{MI}C_{A\;+\;P}$ and $\mathrm{MI}C_{P\;+\;A}$ is the MIC of drug in presence of phage and the MIC of phage in presence of drug at iso-effective combinations (>90% inhibition), respectively. To capture synergistic and antagonistic interactions, the FICmin and FICmax were calculated for each strain and each replicate for all antibiotics tested.^15^ Synergy, additivity and antagonism were considered when the median FICi of the three independent replicates were ≤0.5, between 0.5–4 and >4, respectively.^16^

### Clinical significance of interactions

To assess the clinical relevance of *in vitro* interactions, the percentage of strains where the interactions were at or lower than clinically achievable free drug concentrations in human blood after the administration of established doses of the drugs were calculated, i.e. *f*C_max_ − *f*C_min_ 2.2–0.91 mg/L colistin,^17^ 66.99–0.93 mg/L amikacin,^18^ 49–0.2 mg/L meropenem,^19^ 76.84–34.34/13.4–7.8 mg/L ceftazidime/avibactam^20^ and 2.88–0.31 mg/L ciprofloxacin,^21^ were calculated. For phages, blood levels ranged from 4 × 10^3^ to 1.8 × 10^4^ PFU/mL after intravenous administration of 4 × 10^9^ PFU q8h.^22^

The percentage of strains whose phenotypic resistance was reversed was defined as the number of resistant strains that in the presence of the phages became susceptible, i.e. $\mathrm{MI}C_{A\;+\;P}$ ≤ 2 mg/L for colistin,  ≤ 8 mg/L for amikacin,  ≤ 2 mg/L for meropenem,  ≤ 8 mg/L for ceftazidime/avibactam,  ≤ 0.25 mg/L for ciprofloxacin and or susceptible increased exposure, i.e. $\mathrm{MI}C_{A\;+\;P}$ ≤ 8 mg/L for meropenem.^23^ The reversal of phenotypic resistance was calculated only for resistant isolates based on the MIC_A_ and the $\mathrm{MI}C_{A\;+\;P}$ for synergistic, additive and antagonistic interactions. The susceptible isolates (seven for amikacin and six for colistin) were excluded from this analysis.

### Emergence of resistance

To study the emergence of resistance, the most promising combination, was studied in growth curves and time–kill assays (TKA) at concentrations where synergy was found. Application of single phage as well as cocktail of four phages at different PFU/mL and antibiotic concentrations used in the chequerboard assay was studied with growth curves and TKA to assess growth inhibitory and bactericidal interactions. A positive growth control (bacteria without phages) as well as a negative control (LB + medium) were also included. (i) For growth-curve experiments, 96-well plates were incubated inside a spectrophotometer (Infinite M200, Tecan) and the OD_600_ was measured every 30 min for 48 h using orbital shaking (220 rpm) for 10 s. Growth curves to study emergence of resistance were obtained by plotting OD_600_ measurements for both single agents and combinations in different time intervals using GraphPad Prism v.8.0.2. (ii) For time–kill experiments, to assess the bactericidal effect of phages alone and in a cocktail in combination with antibiotic, samples of 20 μL were taken from each well at selected time intervals (0, 8, 24, 32 and 48 h after the start of the experiment) and serial 10-fold dilutions in 0.9% saline solution in 96-well plates were prepared. Ten microlitres from each dilution and an undiluted sample were plated on Mueller–Hinton agar plates (BD Bioscience, Athens, Greece). The cfu were counted after incubation for 20–24 h at 37 °C. The lower limit of detection was 33.3 cfu/mL per plate, corresponding to 1.52 log_10_ cfu/mL. The absence of growth after 48 h was regarded as complete kill. At 48 h resistance of regrown isolates was verified by determining their MIC_A_ and MIC_P_ in parallel with the parental strain. Time–kill experiments of phage–antibiotic combinations using cocktails of four phages were also performed as phages are usually administered in patients as phage cocktails and phage–phage interactions can either be synergistic or antagonistic.^24^

## Results

### In vitro *activity of drugs and phages alone and in combination*

The EUCAST MICs of all drugs and phages used against each MDR CRKP strain are shown in Table 1. All strains were resistant to the antibiotics tested, except 6/15 (40%) and 7/15 (47%) strains that were susceptible to colistin and amikacin, respectively. The number of strains with synergistic, additive and antagonistic interactions together with the median (range) FICi, MIC_A_ and MIC_P_ alone and in combination, are shown in Table 2 for each drug. Synergy was found in 6/15 strains (40%) for ciprofloxacin, 10/15 (67%) for meropenem, 11/15 (73%) for ceftazidime/avibactam, 8/15 (53%) for colistin and 9/15 (60%) for amikacin reducing the median MIC_A_s by 3–7 2-fold dilutions and median MIC_P_ by 0–1 10-fold dilution. Additivity was observed for the remaining strains. For meropenem and ceftazidime/avibactam combinations, 1/15 (6%) isolates and for ciprofloxacin 2/15 (13%) showed antagonistic effects because MIC_P_ increased by 1 10-fold dilution without increasing the MIC_A_ of drugs. The synergistic interactions were strong as the median (range) FICi was 0.07 (0.01–0.11) for ciprofloxacin, 0.14 (0.04–0.35) for meropenem, 0.10 (0.01–0.22) for ceftazidime/avibactam, 0.10 (0.01–0.22) for colistin and 0.22 (0.10–0.26) for amikacin.

**Table 1.** Median (range) minimum inhibitory phage concentrations (MIC_P_) and minimum inhibitory concentrations (MIC_A_) of different antibiotics against 15 MDR carbapenemase-producing *K. pneumoniae* clinical isolates

Isolates	Resistance mechanisms	MLST	Capsular type	Phages	MIC_P_ (PFU/mL)	MIC_A_ (mg/L)				
						Colistin	Meropenem	Ciprofloxacin	Amikacin	Ceftazidime/ Avibactam
AUHB130	KPC + VIM	ST147	K64	vB_KpP_AttikonH1	5 × 10^4^ (5 × 10^3^–5 × 10^4^)	8 (4–8)	256 (128–256)	256 (256−>256)	8 (4–16)	>64 (>64−>64)
AUHB57	KPC + VIM	ST147	K64	vB_KpP_AttikonH1	5 × 10^3^ (5 × 10^0^–5 × 10^5^)	2 (2–4)	64 (64–64)	32 (32–32)	8 (8–16)	>64 (>64−>64)
AUHB68	KPC + VIM	Uknown	K64	vB_KpP_AttikonH1	5 × 10^3^ (5 × 10^1^–5 × 10^5^)	2 (2–2)	64 (64–128)	256 (256–256)	4 (4–4)	>64 (>64−>64)
AUHB133	VIM	ST147	K64	vB_KpP_AttikonH1	5 × 10^8^ (5 × 10^5^–5 × 10^9^)	4 (4–8)	128 (64–128)	256 (256–256)	8 (8–16)	16 (16–16)
AUHB24	VIM	ST323	K21	vB_KpP_AttikonH2	5 × 10^2^ (5 × 10^1^–5 × 10^3^)	2 (2–2)	64 (32–128)	16 (16–16)	256 (256–256)	>64 (>64−>64)
AUHB204	KPC + VIM	ST147	K64	vB_KpP_AttikonH2	5 × 10^7^ (5 × 10^7^–5 × 10^9^)	>8 (>8−>8)	>256 (>256−>256)	256 (256–256)	8 (8–16)	>64 (>64−>64)
AUHB73	VIM	ST147	K64	vB_KpP_AttikonH2	5 × 10^0^ (5 × 10^0^–5 × 10^2^)	2 (2–2)	128 (128–256)	>256 (256−>256)	8 (2–16)	>64 (>64−>64)
AUHB209	KPC + VIM	ST323	K50	vB_KpP_AttikonH2	5 × 10^2^ (5 × 10^2^–5 × 10^4^)	>8 (>8−>8)	>256 (>256−>256)	16 (16–16)	256 (256–256)	>64 (>64−>64)
AUHB135	NDM	ST11	K24	vB_KpP_AttikonH4	5 × 10^0^ (5 × 10^0^–5 × 10^1^)	2 (2–4)	32 (32–64)	>256 (256−>256)	4 (2–4)	>64 (>64−>64)
AUHB138	VIM	ST11	K24	vB_KpP_AttikonH4	5 × 10^1^ (5 × 10^0^–5 × 10^1^)	8 (8−>8)	32 (32–32)	>256 (>256−>256)	256 (256–256)	64 (32−>64)
AUHB141	NDM	ST11	K24	vB_KpP_AttikonH4	5 × 10^0^ (5 × 10^0^–5 × 10^3^)	2 (2–2)	32 (32–64)	256 (256–256)	16 (8–16)	>64 (>64−>64)
AUHB114	VIM	ST11	K24	vB_KpP_AttikonH5	5 × 10^1^ (5 × 10^0^–5 × 10^2^)	>8 (8−>8)	32 (32–64)	256 (256–256)	64 (32–256)	>64 (>64−>64)
AUHB129	NDM	ST11	K24	vB_KpP_AttikonH5	5 × 10^1^ (5 × 10^0^–5 × 10^1^)	4 (2–4)	256 (256–256)	256 (>64–256)	16 (8–16)	>64 (>64−>64)
AUHB7	NDM	ST11	K24	vB_KpP_AttikonH5	5 × 10^3^ (5 × 10^0^–5 × 10^4^)	>8 (>8−>8)	32 (32–64)	256 (256–256)	16 (16–16)	>64 (>64−>64)
AUHB127	NDM	ST11	K24	vB_KpP_AttikonH5	5 × 10^1^ (5 × 10^0^–5 × 10^2^)	8 (8−>8)	64 (32–64)	>256 (256−>256)	16 (8–16)	>64 (>64−>64)

**Table 2.** Median (range) MIC of five antibiotics (MIC_A_) and four phages (MIC_P_) alone and in synergistic, additive, and antagonistic combinations, as well as the fractional inhibitory concentration index (FICi) against 15 MDR carbapenemase-producing *K. pneumoniae* clinical isolates using the chequerboard assay

Antibiotic (No. of strains)	Interactions	FICi ^a^	Agents alone		Agents in combination	
	(No. of strains)^a^		MIC_A_ (mg/L)	MIC_P_ (PFU/mL)	MIC_A+P_ (mg/L)	MIC_P+A_ (PFU/mL)
Colistin (15)	Synergistic (8)^1,2,3,4^	0.10 (0.01–0.22)	8 (2−>8)	5 × 10^3^ (5 × 10^1^–5 × 10^7^)	0.125 (0.125–0.25)	5 × 10^2^ (5 × 10^0^–5 × 10^6^)
	Additive (7)^1,2,3,4^	2.01 (1.5–2.1)	2 (2−>8)	5 × 10^0^ (5 × 10^0^–5 × 10^1^)	2 (0.125–4)	5 × 10^1^ (5× 10^0^–5 × 10^2^)
	Antagonistic (0)	NA^b^	NA	NA	NA	NA
Ciprofloxacin (15)	Synergistic (6)^1,2,3,4^	0.07 (0.01–0.11)	256 (256−>256)	5 × 10^1^ (5 × 10^1^–5 × 10^8^)	4 (4–8)	5 × 10^0^ (5 × 10^0^–5 × 10^6^)
	Additive (7)^1,2,3,4^	1.5 (1.25–1.5)	256 (16−>256)	5 × 10^2^ (5 × 10^0^–5 × 10^4^)	128 (4–256)	5 × 10^3^ (5 × 10^0^–5 × 10^5^)
	Antagonistic (2)^3,4^	10.5/10.25	256/>256)	5 × 10^1^/5 × 10^0^	128/128	5 × 10^2^/5 × 10^1^
Meropenem (15)	Synergistic (10)^1,2,3,4^	0.14 (0.04–0.35)	128 (32−>256)	5 × 10^2^ (5 × 10^1^–5 × 10^9^)	8 (1–128)	5 × 10^1^ (5 × 10^0^–5 × 10^5^)
	Additive (4)^2,3,4^	1.25 (1.25–1.5)	32 (32–128)	5 × 10^0^ (5 × 10^0^–5 × 10^9^)	32 (16–128)	5 × 10^1^ (5 × 10^0^–5 × 10^6^)
	Antagonistic (1)^4^	11	32	5 × 10^0^	32	5 × 10^1^
Ceftazidime/avibactam (15)	Synergistic (11)^1,2,3,4^	0.10 (0.01–0.22)	>64 (16−>64)	5 × 10^2^ (5 × 10^1^–5 × 10^8^)	2 (1–16)	5 × 10^1^ (5 × 10^0^–5 × 10^6^)
	Additive (3)^1,2,3^	1.5 (1.5–1.5)	>64 (>64−>64)	5 × 10^0^ (5 × 10^0^–5 × 10^3^)	1 (1–2)	5 × 10^1^ (5 × 10^0^–5 × 10^3^)
	Antagonistic (1)^3^	10.5	>64	5 × 10^3^	64	5 × 10^4^
Amikacin (15)	Synergistic (9)^1,2,3,4^	0.22 (0.10–0.26)	16 (8–256)	5 × 10^3^ (5 × 10^1^–5 × 10^7^)	2 (2–4)	5 × 10^2^ (5 × 10^0^–5 × 10^5^)
	Additive (6)^1,2,3,4^	1.75 (1.5–2.1)	16 (4–256)	5 × 10^1^ (5 × 10^0^–5 × 10^3^)	8 (2–8)	5 × 10^1^ (5 × 10^0^–5 × 10^2^)
	Antagonistic (0)	NA	NA	NA	NA	NA

^a^1, vB_KpP_AttikonH1; 2, vB_KpP_AttikonH2; 3, vB_KpP_AttikonH4; 4, vB_KpP_AttikonH5.

^b^NA, not applicable.

### Clinical relevance of interactions

**Figure 2.**
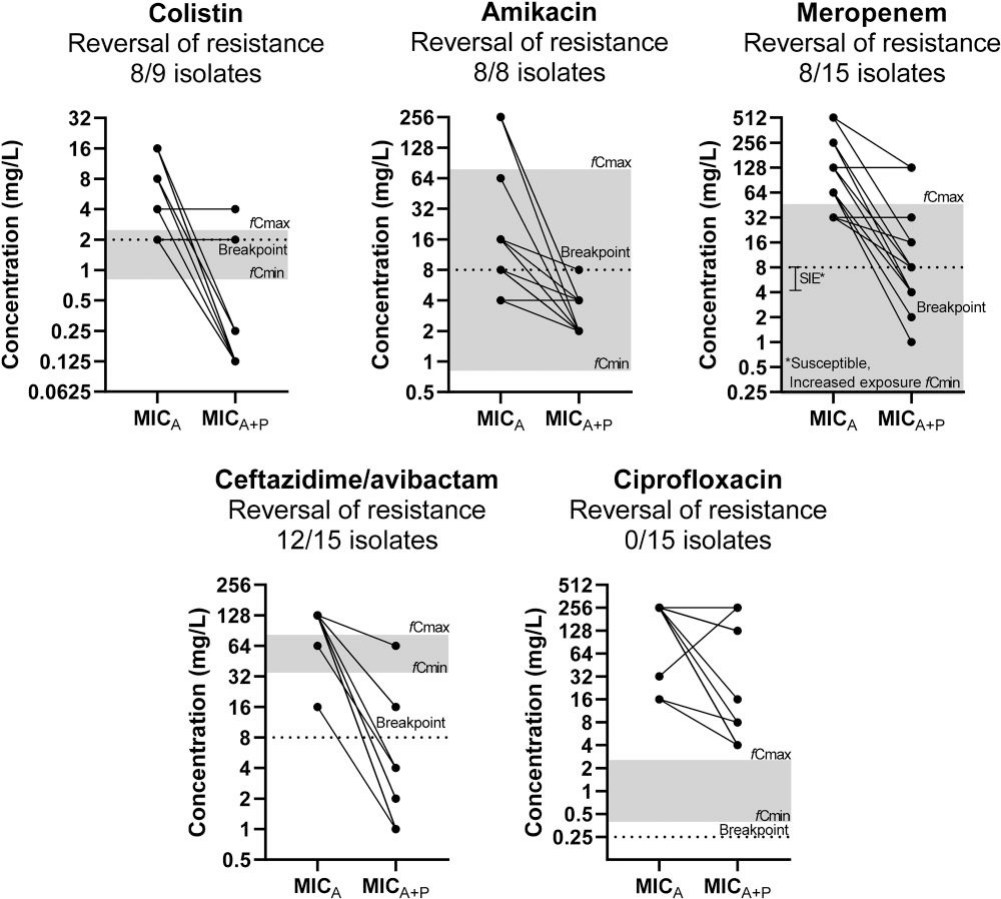
Effect of phages on MIC of antibiotics (MIC_A_) in relation to clinically achievable serum concentrations and clinical breakpoints (reversal of phenotypic resistance) for resistant to antibiotics *K. pneumoniae* isolates. The MIC_A_ of each isolate is presented on the left and the MIC_A_ in combination with the phage ($\mathrm{MI}C_{A\;+\;P}$) on the right. Each line may represent more than one isolate. The grey zone represents the area of clinically achievable free drug concentrations in human blood (*fC*_max_ − *fC*_min_). The dotted line represents the EUCAST breakpoint of each drug.

Reversal of phenotypic resistance was observed in 8/8 (100%) strains for amikacin, 8/9 (89%) for colistin and 12/15 (80%) for ceftazidime/avibactam based on EUCAST clinical breakpoints (Figure 2). Regarding meropenem, among the 8/15 (53%) of isolates that reversal of phenotypic resistance was observed, 2/8 (25%) became susceptible and 6/8 (75%) were categorized as susceptible, increased exposure. For phages and for most antibacterial agents tested, synergistic interactions were found at clinically achievable concentrations, except for ciprofloxacin. Regarding antibiotics, clinically achievable concentrations, after combination with phages, were observed for amikacin, meropenem, colistin and ceftazidime/avibactam in 15/15 (100%), 13/15 (87%), 14/15 (93%) and 15/15 (100%) strains, respectively (Figure 2). For phages, clinically achievable serum concentrations, after combination with antibiotics, were observed in 13/15 (87%) of isolates for amikacin, meropenem and ceftazidime/avibactam and in 12/15 (80%) of isolates for colistin and ciprofloxacin.

### Emergence of resistance

**Figure 3.**
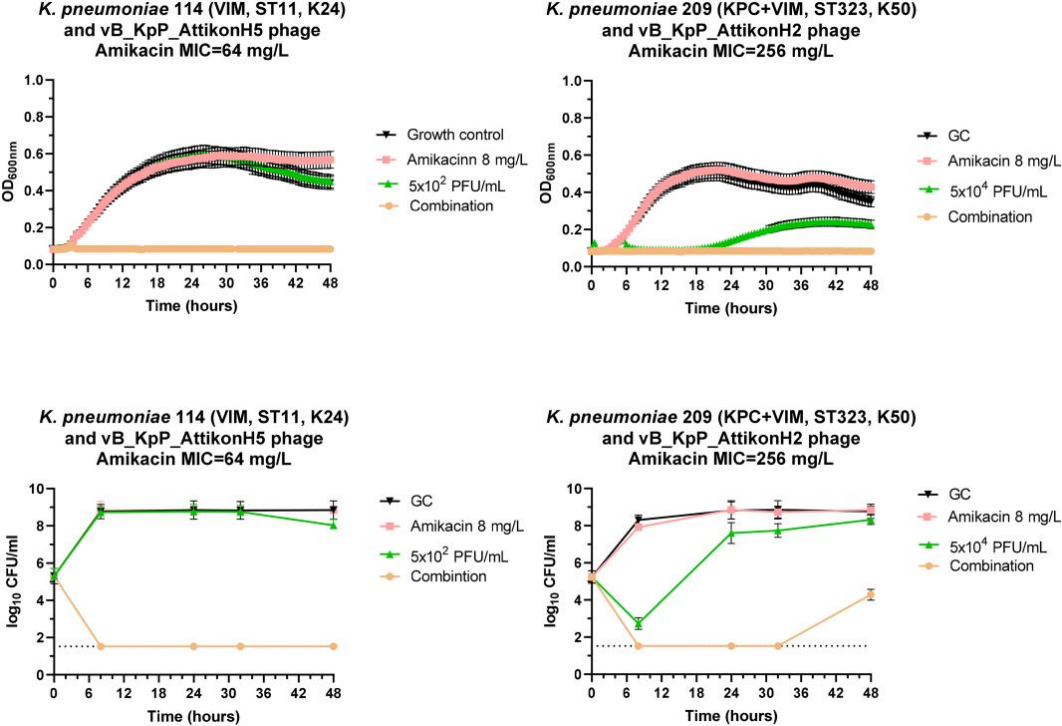
Growth curves (top) and time–kill curves (bottom) of amikacin and phage alone and in combination using two different phages against a VIM and KPC + VIM producing *K. pneumoniae* isolates. For growth curves, emergence of resistance was monitored using spectrophotometer with measurements every 30 minutes for 48 h. For time–kill curves, log_10_ cfu/ml at different time intervals were plotted through 48 h. Error bars indicate SDs.

**Figure 4.**
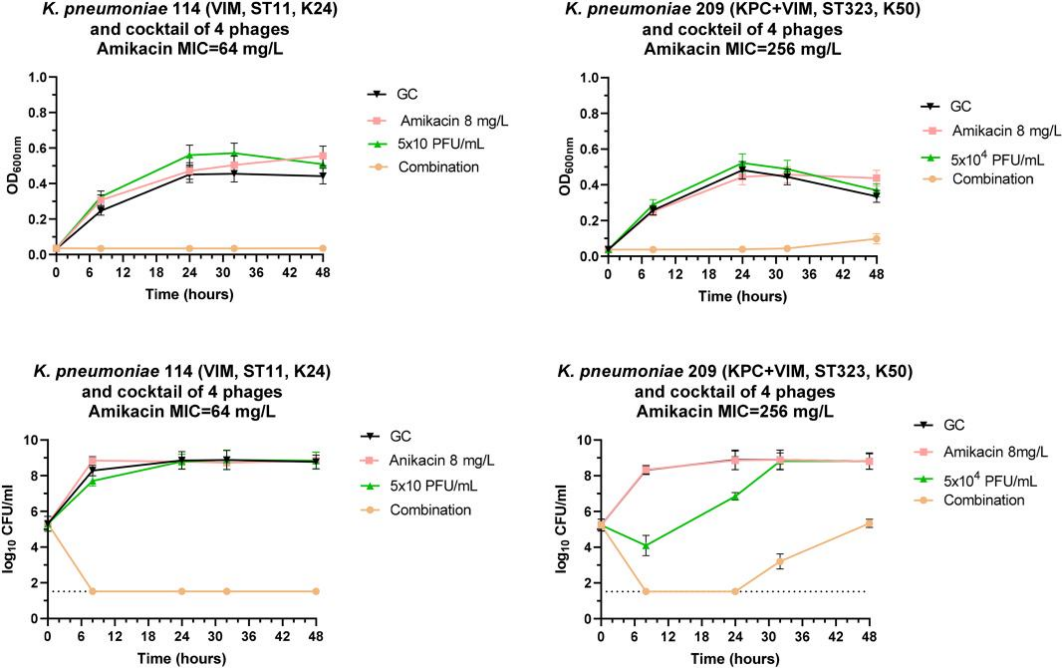
Growth curves (top) and time–kill curves (bottom) of amikacin alone and in combination with a cocktail of four different phages against a VIM and KPC + VIM producing *K. pneumoniae* isolates. For growth curves, emergence of resistance was monitored using spectrophotometer with measurements every 30 minutes for 48 h. For time–kill curves, log_10_ cfu/ml at different time intervals were plotted through 48 h. Error bars indicate SDs.

The emergence of resistance against amikacin alone and in combination with phages alone and in cocktails was studied with growth curves and TKA against 2 CRKP isolates with different resistant mechanisms to carbapenems (VIM and VIM + KPC) and low- and high-level resistance to amikacin with MICs 64 and 256 mg/L, respectively. Growth-curve experiments showed that the combination of amikacin with single phages at breakpoint concentration of 8 mg/L and down to phage concentrations of 5 × 10^2^ PFU/mL where the synergistic interactions were found inhibited growth for 48 h (Figure 3). Time–kill experiments confirmed synergistic interactions, although for the *K. pneumoniae* isolate with the high MIC to amikacin emergence of resistance was found after 24 h of incubation. Similar results were found when amikacin was combined with a cocktail of phages (Figure 4). The MIC_p_ of bacteria emerged in combination therapy were 10^3^- and 10^5^-fold higher for AUHB114 and AUHB209 than the initial MIC_p_, respectively. No differences were found for the MIC of amikacin.

## Discussion

Synergistic interactions were found between phages and antibiotics of different classes with most synergistic combination found for ceftazidime/avibactam (73% of isolates) followed by meropenem (67% of isolates), amikacin (60% of isolates), colistin (53% of isolates) and ciprofloxacin (40% of isolates). Synergistic interactions were strong as the median (range) FICi were 0.07 (0.01–0.11) for ciprofloxacin, 0.10 (0.01–0.22) for ceftazidime/avibactam, 0.10 (0.01–0.22) for colistin, 0.14 (0.04–0.35) for meropenem and 0.22 (0.10–0.26) for amikacin. For all drug–phage combinations, except ciprofloxacin, clinically achievable concentrations were found for both phages and antibiotics whereas the MIC_A_ decreased at or below the susceptibility breakpoint for most resistant isolates for amikacin (100%), colistin (89%) and ceftazidime/avibactam (80%) and less for meropenem (53%). Inhibition of bacterial growth was observed up to 48 h and time–kill experiments showed extensive killing of amikacin–phage combinations compared to single agents for isolates with low MIC to amikacin (MΙC 64 mg/L), whereas for isolates with high MIC to amikacin (MΙC 256 mg/L) emergence of resistance was observed after 24 h despite the extensive killing at 0–24 h. Interactions were not affected by the cocktail of phages.

Synergistic interactions between phages and antibiotics was explained by a number of mechanisms, including: antibiotic-induced production of phages by bacterial hosts,^25^ antibiotic-induced cell elongation, accelerated phage amplification and enhanced burst size, reduction of phage and/or antibiotic-resistant mutant appearance, increased antibiotic susceptibility as a result of the presence of the phage, decreased antibiotic MIC following the addition of phages to an antibiotic as presented in the current study and depolymerization of the bacterial polysaccharides by phage enzymes.^25^ On the other hand, antagonistic interactions can occur via side phage–receptor site sharing or abortive infection pathways,^15^ blocking an early step of the phage life cycle before DNA replication by aminoglycosides,^26^ inhibition of DNA gyrase/topoisomerase as part of the host machinery co-opted by the phage for replication by quinolones, reduction of bacterial growth rates by bacteriostatic antibiotics promoting the evolution of CRISPR immunity,^27^ and disruption of anti-CRISPR proteins Acr by translation inhibitors re-sensitizing phages to complete CRISPR–Cas immunity.^28^ However, interactions may be affected by specific phages and antibiotics used, drug and phage concentrations, and experimental design and conditions (sequence of drug + phage application, exposure period, host and *in vitro* environment).^29^

Meropenem and ceftazidime/avibactam–phage combination were synergistic for most isolates compared to other antibiotic-phage combinations. As meropenem and ceftazidime/avibactam are both β-lactams binding to penicillin-binding proteins in the bacterial cell wall and inhibiting peptidoglycan, it seems that β-lactam-phage combinations may be superior to other antibiotic-phage combinations. Furthermore, although similar FICis were found and synergy was observed for ceftazidime/avibactam and colistin at clinically achievable concentrations for most strains, reversal of phenotypic resistance was found for more isolates for ceftazidime/avibactam. If there is no mechanistic difference between interaction of cephalosporins and carbapenems with phages, this difference may be due to the presence of β-lactamase inhibitor avibactam that can protect ceftazidime from degradation by β-lactamases.^30,31^ The fact that synergistic interactions did occur at phage concentrations previously described in human blood makes the ceftazidime/avibactam–phage combination even more appealing.^32^

For amikacin + phage combination, it was observed both reversal of phenotypic resistance for all resistant isolates and prevention of emergence of resistance to phages as verified in growth curve and time–kill experiments for isolates with low MIC to amikacin. For isolates with high MIC despite the extensive killing by phages alone in the first 24 h, resistant isolates emerged after 24 h. The clinical relevance of this phage resistance is of unknown but is expected not to be of major importance as emerged phage-resistant colonies are usually avirulent.^33^ Furthermore, such resistant clones emerged *in vitro* also during drug exposure^34^ and is believed that is restricted *in vivo* and therefore the PK/PD driver is usually associated with stasis/1log kill without considering emergence of resistance.

Our results were in concordance with previous findings from other *in vitro* and *in vivo* experiments using combination therapy of phages and antibiotics against *K. pneumoniae* isolates. In an *in vivo* study, researchers reported that using imipenem in combination with phages against clinical isolates of *K. pneumoniae* significantly increased the survival rate of *Galleria mellonella* model by 75% compared to phage monotherapy.^35^ In another study combination of phage with low dosages of meropenem led to an entire bacterial eradication, evident by the lack of cultivable bacteria at the end of the TKA.^36^ Phage-amikacin combination exhibited potent synergistic activities *in vitro* for *K. pneumoniae* isolates by substantially decreasing their growth and viabilities within 2 h, and completely suppressing bacterial regrowth until 24 h.^32^ These findings were also confirmed in an *in vivo* murine model of bacteraemia where phage + amikacin combination therapy significantly reduced bacterial burden and improved murine survival to 100% by Day 7.^32^ Similar results have been found also in an *in vivo G. mellonella* model with 80% survival after phage + amikacin combination therapy at 4 days against an MDR *K. oxytoca* isolate.^37^ A chequerboard analysis similar to the one used in the present study indicated that when meropenem and amikacin were used in combination with a single phage, against CRKP, FICi values were <0.5 and MIC of these antibiotics were significantly reduced from 128 and >2048 mg/L to 16 and 1024 mg/L respectively.^38^ Similar results have been found and for ceftazidime/avibactam in combination with phages were in a neutropenic murine model of gastrointestinal translocation with a CRKP isolate, treatment of mice with 5 × 10^9^ PFU of phage cocktail was effective in significantly reducing renal CFU by 100-fold when administered every 24 h and 1000-fold every 12 h.^39^

However, it was previously found that antibiotics targeting bacterial protein synthesis (e.g. aminoglycosides) reduced the lytic activity of bacteriophage against *Acinetobacter baumannii*, *Staphylococcus aureus* and *Salmonella typhimurium*.^26,40,41^ None of these three studies have tested *K. pneumoniae* isolates and other than broth microdilution chequerboard methods were used to study the interactions capturing early events (9 h in growth curves assay) or low concentration effects (plaque size outside to inhibition zone) or using non-cation-adjusted media. MgCl_2_ was previously found that phage infection was completely restored by the addition of as evidenced by the strong growth defect and the increasing phage titre during infection.^26^

The chequerboard broth microdilution method is the standard approach for evaluating drug combinations by estimating the reduction in MIC_A_ in combination relative to the MIC_A_s of the antibiotics alone.^42^ When both antibacterial agents’ MIC_A_s are lowered by more than two 2-fold dilutions, then FICi is ≤0.5 and synergy is concluded. Considering that, we used the same approach to evaluate phage–antibiotic interactions. However, the FICi of 0.5 was proposed to detect significant microbiological synergy based on 2-fold variation that usually the MICs of antibiotics exhibit when determined using 2-fold serial dilutions. As phages are usually diluted 10-fold, an adjustment of FIC index cutoffs may be needed. Furthermore, as the FICi is the sum of two concentration ratios (FIC_A_ and FIC_B_), information on which compound is the effector and which is the affected is lost when the two ratios are combined.^43^ Hence, we explored the effect of phages on MIC of antibiotics and vice versa, irrespective of FIC index. In addition, interactions are assessed only at the MIC levels without taking to account the entire response surface. As interactions at the MIC level are expected to be more clinically significant and since the sub-MIC effects were minimal, FICis at the MIC levels captured the most important interactions between phages and antibiotics.

Furthermore, the MIC_A_ reduction could be clinically irrelevant if it happens at supraphysiological concentrations, or if MIC_A_s are not reduced below the susceptibility breakpoint. All antibiotics included showed comparable FICis and showed synergy at clinically achievable free drug concentrations. However, amikacin was found to reverse phenotypic resistance in all strains, making it the best candidate for phage + antibiotic combination against CRKP isolates. Although the strongest synergy (lowest FICis) was found with ciprofloxacin, synergistic concentrations were observed at clinically unachievable concentrations. Such analysis cannot be done for phage concentrations as there is no critical threshold concentrations or breakpoints correlated with clinical outcome. Finally, host factors such as serum and phagocytes that neutralize phage particles have was not studied. However, the fact that *in vitro* synergistic effects in broth microdilution chequerboard experiments were verified in animal models^32^ increases the *in vitro* translatability of the findings of the present study.

In the present study, *in vitro* changes of MICs of antibiotics were lowered during co-exposure to phage at clinical achievable concentrations. It is uncertain whether such MIC shifts will be observed *in vivo* as in a well plate, there is extensive phage replication leading to local very high phage concentrations, whereas in the clinical setting phage concentrations are variable and often quickly cleared after administration. Animal and clinical data showed that phages are distributed to different organs after intra- and extra-vascular administration.^3^ Following intravenous bolus administration of 4 × 10^9^ PFU of bacteriophages, the phage concentration was 1.8 × 10^4^ PFU/ml at 5 min and undetectable at 60 min in human serum.^22^ However, phages may replicate at the site of infection as found in a patient with MDR *P. aeruginosa* pneumonia treated intravenously pre-fixed bacteriophage cocktail (4 × 10^9^ PFU q6h) for 1 week where no viable bacteriophages were detected in serum drawn within 30 min of the next dose, while ∼4 × 10^7^ PFU/mL was recovered in BAL obtained 3 days after initiation of bacteriophage therapy.^44^ In addition, despite the fast clearance from serum, there are multiple reports of successful outcome in patients with deep infections treated intravenously with phages.^3^

In conclusion, synergistic interactions were found using phages in combination with various classes of antibiotics. Among all the antibiotics studied, we consider phage combination therapy with amikacin to be the most promising, as drug concentrations in the combination were within clinically achievable blood concentrations and there was reversal of phenotypic resistance for all strains. Further studies are needed in a dynamic model where phage and antibiotic human pharmacokinetics will be simulated and in animal models where host-related factors will be considered. While randomized clinical trial data are still pending and several questions on phage + antibiotic combination therapy are unanswered such as the PK/PD driver, optimal dosing regimens, tissue penetration, impact of serum on synergistic interactions, *in vitro* studies of phage + antibiotic combinations are essential for expanding our knowledge of possible interactions. Our results support the use of phages in combination with antibiotics against MDR strains specifically of CPKPs where therapeutic options are not available.

## References

[1] Ramos-Castañeda JA, Ruano-Ravina A, Barbosa-Lorenzo R et al Mortality due to KPC carbapenemase-producing *Klebsiella pneumoniae* infections: systematic review and meta-analysis: mortality due to KPC *Klebsiella pneumoniae* infections. J Infect 2018; 76: 438–48. 10.1016/j.jinf.2018.02.007 29477802

[2] European Centre for Disease Prevention and Control . Antimicrobial Resistance in the EU/EEA (EARS-Net)—Annual Epidemiological Report 2022. ECDC, 2023.

[3] Siopi M, Skliros D, Paranos P et al Pharmacokinetics and pharmacodynamics of bacteriophage therapy: a review with a focus on multidrug-resistant Gram-negative bacterial infections. Clin Microbiol Rev 2024; 37: e00044-24. 10.1128/cmr.00044-24 39072666 PMC11391690

[4] Kortright KE, Chan BK, Koff JL et al Phage therapy: a renewed approach to combat antibiotic-resistant bacteria. Cell Host Microbe 2019; 25: 219–32. 10.1016/j.chom.2019.01.014 30763536

[5] Oechslin F . Resistance development to bacteriophages occurring during bacteriophage therapy. Viruses 2018; 10: 351. 10.3390/v10070351 29966329 PMC6070868

[6] Tagliaferri TL, Jansen M, Horz H-P. Fighting pathogenic bacteria on two fronts: phages and antibiotics as combined strategy. Front Cell Infect Microbiol 2019; 9: 429985. 10.3389/fcimb.2019.00022 PMC6387922 30834237

[7] Torres-Barceló C, Hochberg ME. Evolutionary rationale for phages as complements of antibiotics. Trends Microbiol 2016; 24: 249–56. 10.1016/j.tim.2015.12.011 26786863

[8] Meletiadis J, Paranos P, Georgiou P-C et al In vitro comparative activity of the new beta-lactamase inhibitor taniborbactam with cefepime or meropenem against *Klebsiella pneumoniae* and cefepime against *Pseudomonas aeruginosa* metallo-beta-lactamase-producing clinical isolates. Int J Antimicrob Agents 2021; 58: 106440. 10.1016/j.ijantimicag.2021.106440 34551356

[9] Larsen MV, Cosentino S, Rasmussen S et al Multilocus sequence typing of total-genome-sequenced bacteria. J Clin Microbiol 2012; 50: 1355–61. 10.1128/JCM.06094-11 22238442 PMC3318499

[10] Lam MMC, Wick RR, Judd LM et al Kaptive 2.0: updated capsule and lipopolysaccharide locus typing for the *Klebsiella pneumoniae* species complex. Microb Genom 2022; 8: 000800. 10.1099/mgen.0.000800 35311639 PMC9176290

[11] Paranos P, Skliros D, Zrelovs N et al Isolation and characterization of lytic bacteriophages against carbapenemase-producing *K. pneumoniae* clinical isolates. *Thirty-third European Congress of Clinical Microbiology & Infectious Diseases of Clinical Microbiology & Infectious Diseases, Copenhagen, Denmark, 2023*. Abstract 03143.

[12] Paranos P, Pournaras S, Meletiadis J. A single-layer spot assay for easy, fast, and high-throughput quantitation of phages against multidrug-resistant Gram-negative pathogens. J Clin Microbiol 2024; 62: e00743-24. 10.1128/jcm.00743-24 39072625 PMC11323465

[13] CLSI . Methods for Dilution Antimicrobial Susceptibility Tests for Bacteria That Grow Aerobically—Twelfth Edition: M07. 2024.

[14] Agún S, Fernández L, González-Menéndez E et al Study of the interactions between bacteriophage phiIPLA-RODI and four chemical disinfectants for the elimination of *Staphylococcus aureus* contamination. Viruses 2018; 10: 103. 10.3390/v10030103 29495568 PMC5869496

[15] Paranos P, Vourli S, Pournaras S et al In vitro interactions between bacteriophages and antibacterial agents of various classes against multidrug-resistant metallo-β-lactamase-producing *Pseudomonas aeruginosa* clinical isolates. Pharmaceuticals 2025; 18: 343. 10.3390/ph18030343 40143121 PMC11945160

[16] Odds FC . Synergy, antagonism, and what the chequerboard puts between them. J Antimicrob Chemother. 2003; 52: 1. doi:10.1093/jac/dkg301 12805255

[17] Kim EJ, Oh J, Lee K et al Pharmacokinetic characteristics and limited sampling strategies for therapeutic drug monitoring of colistin in patients with multidrug-resistant Gram-negative bacterial infections. Ther Drug Monit 2019; 41: 102–6. 10.1097/FTD.0000000000000572 30299430

[18] European Committee on Antimicrobial Susceptibility Testing . Amikacin: rationale for the clinical breakpoints. version 3.0. 2024. http://www.eucast.org.

[19] European Committee on Antimicrobial Susceptibility Testing . Meropenem: rationale for the clinical breakpoints. version 3.0. 2024. http://www.eucast.org.

[20] Kline EG, Nguyen MHT, McCreary EK et al 1298. population pharmacokinetics of ceftazidime-avibactam among critically-ill patients with and without receipt of continuous renal replacement therapy. Open Forum Infect Dis 2020; 7: S663–4. 10.1093/ofid/ofaa439.1481

[21] Szałek E, Kamińska A, Gozdzik-Spychalska J et al The PK/PD index (CMAX/MIC) for ciprofloxacin in patients with cystic fibrosis. Acta Pol Pharm 2011; 68: 777–83. 21928725

[22] Schooley RT, Biswas B, Gill JJ et al Development and use of personalized bacteriophage-based therapeutic cocktails to treat a patient with a disseminated resistant *Acinetobacter baumannii* infection. Antimicrob Agents Chemother 2017; 61: e00954-17. 10.1128/AAC.00954-17 28807909 PMC5610518

[23] The European Committee on Antimicrobial Susceptibility Testing . Breakpoint tables for interpretation of MICs and zone diameters. Version 14.0. 2024. http://www.eucast.org.

[24] Kim MK, Chen Q, Echterhof A et al A blueprint for broadly effective bacteriophage-antibiotic cocktails against bacterial infections. Nat Commun 2024; 15: 9887. 10.1038/s41467-024-53994-9 39609398 PMC11604943

[25] Łusiak-Szelachowska M, Międzybrodzki R, Drulis-Kawa Z et al Bacteriophages and antibiotic interactions in clinical practice: what we have learned so far. J Biomed Sci 2022; 29: 23. 10.1186/s12929-022-00806-1 35354477 PMC8969238

[26] Kever L, Hardy A, Luthe T et al Aminoglycoside antibiotics inhibit phage infection by blocking an early step of the infection cycle. mBio 2022; 13: e0078322. 10.1128/mbio.00783-22 35506667 PMC9239200

[27] Dimitriu T, Kurilovich E, Łapińska U et al Bacteriostatic antibiotics promote CRISPR-Cas adaptive immunity by enabling increased spacer acquisition. Cell Host Microbe 2022; 30: 31–40.e5. 10.1016/j.chom.2021.11.014 34932986

[28] Pons BJ, Dimitriu T, Westra ER et al Antibiotics that affect translation can antagonize phage infectivity by interfering with the deployment of counter-defenses. Proc Natl Acad Sci U S A 2023; 120: e2216084120. 10.1073/pnas.2216084120 36669116 PMC9942909

[29] Ma D, Li L, Han K et al The antagonistic interactions between a polyvalent phage SaP7 and β-lactam antibiotics on combined therapies. Vet Microbiol 2022; 266: 109332. 10.1016/j.vetmic.2022.109332 35033842

[30] Dhillon S . Meropenem/vaborbactam: a review in complicated urinary tract infections. Drugs 2018; 78: 1259. 10.1007/s40265-018-0966-7 30128699 PMC6132495

[31] Mosley JF, Smith LL, Parke CK et al Ceftazidime-avibactam (avycaz): for the treatment of complicated intra-abdominal and urinary tract infections. P T 2016; 41: 479. doi:10.1586/17512433.2015.1090874 27504064 PMC4959616

[32] Shein AMS, Wannigama DL, Hurst C et al Phage cocktail amikacin combination as a potential therapy for bacteremia associated with carbapenemase producing colistin resistant *Klebsiella pneumoniae*. Sci Rep 2024; 14: 28992. 10.1038/s41598-024-79924-9 39578508 PMC11584731

[33] León M, Bastías R. Virulence reduction in bacteriophage resistant bacteria. Front Microbiol 2015; 6: 343. 10.3389/fmicb.2015.00343 25954266 PMC4407575

[34] Meletiadis J, Paranos P, Tsala M et al Pharmacodynamics of colistin resistance in carbapenemase-producing *Klebsiella pneumoniae*: the double-edged sword of heteroresistance and adaptive resistance. J Med Microbiol 2022; 71: 001565. 10.1099/jmm.0.001565 36201344

[35] Pacios O, Fernández-García L, Bleriot I et al Enhanced antibacterial activity of repurposed mitomycin C and imipenem in combination with the lytic phage vB_KpnM-VAC13 against clinical isolates of *Klebsiella pneumoniae*. Antimicrob Agents Chemother 2021; 65: e0090021. 10.1128/AAC.00900-21 34228538 PMC8370222

[36] Ziller L, Blum PC, Buhl EM et al Newly isolated Drexlerviridae phage LAPAZ is physically robust and fosters eradication of *Klebsiella pneumoniae* in combination with meropenem. Virus Res 2024; 347: 199417. 10.1016/j.virusres.2024.199417 38880333 PMC11245953

[37] Zhao Y, Feng L, Zhou B et al A newly isolated bacteriophage vB8388 and its synergistic effect with aminoglycosides against multi-drug resistant *Klebsiella oxytoca* strain FK-8388. Microb Pathog 2023; 174: 105906. 10.1016/j.micpath.2022.105906 36494020

[38] Wang Q, Chen R, Liu H et al Isolation and characterization of lytic bacteriophage vB_KpnP_23: a promising antimicrobial candidate against carbapenem-resistant *Klebsiella pneumoniae*. Virus Res 2024; 350: 199473. 10.1016/j.virusres.2024.199473 39332682 PMC11474366

[39] Zagaliotis P, Michalik-Provasek J, Mavridou E et al Bacteriophage treatment is effective against carbapenem-resistant *Klebsiella pneumoniae* (KPC) in a neutropenic murine model of gastrointestinal translocation and renal infection. Antimicrob Agents Chemother 2024; 69: e00919-24. 10.1128/aac.00919-24 39704532 PMC11823626

[40] Vashisth M, Yashveer S, Anand T et al Antibiotics targeting bacterial protein synthesis reduce the lytic activity of bacteriophages. Virus Res 2022; 321: 198909. 10.1016/j.virusres.2022.198909 36057417

[41] Jiang Z, Wei J, Liang Y et al Aminoglycoside antibiotics inhibit mycobacteriophage infection. Antibiotics 2020; 9: 714. 10.3390/antibiotics9100714 33086520 PMC7603143

[42] Bellio P, Fagnani L, Nazzicone L et al New and simplified method for drug combination studies by checkerboard assay. MethodsX 2021; 8: 101543. 10.1016/j.mex.2021.101543 34754811 PMC8563647

[43] Fatsis-Kavalopoulos N, Sánchez-Hevia DL, Andersson DI. Beyond the FIC index: the extended information from fractional inhibitory concentrations (FICs). J Antimicrob Chemother 2024; 79: 2394–6. 10.1093/jac/dkae233 38997227 PMC11368421

[44] Aslam S, Courtwright AM, Koval C et al Early clinical experience of bacteriophage therapy in 3 lung transplant recipients. Am J Transplant 2019; 19: 2631–9. 10.1111/ajt.15503 31207123 PMC6711787

